# Spatiotemporal Association of Coronavirus Disease 2019 Cases and Deaths With Exposure to Wildfire Particulate Matter in 2020

**DOI:** 10.1093/ofid/ofaf262

**Published:** 2025-06-11

**Authors:** Thomas C McHale, David R Boulware, Kelly Searle, Leda Kobziar, Phinehas Lampman, Julio C Zuniga-Moya, Ben Papadopoulos, Andrej Spec, Naomi E Hauser, George R Thompson

**Affiliations:** Department of Medicine, Division of Infectious Disease and International Medicine, University of Minnesota, Minneapolis, Minnesota, USA; Department of Medicine, Division of Infectious Disease and International Medicine, University of Minnesota, Minneapolis, Minnesota, USA; School of Public Health, Division of Epidemiology & Community Health, University of Minnesota, Minneapolis, Minnesota, USA; Department of Forest, Rangeland and Fire Sciences, University of Idaho, College of Natural Resources, Couer d’Alene, Idaho, USA; Department of Forest, Rangeland and Fire Sciences, University of Idaho, College of Natural Resources, Couer d’Alene, Idaho, USA; Department of Forest, Rangeland and Fire Sciences, University of Idaho, College of Natural Resources, Moscow, Idaho, USA; Department of Internal Medicine, Division of Infectious Diseases, Washington University, St. Louis, Missouri, USA; Department of Internal Medicine, Division of Infectious Diseases, Washington University, St. Louis, Missouri, USA; Department of Internal Medicine, Division of Infectious Diseases, Washington University, St. Louis, Missouri, USA; Department of Internal Medicine, Division of Infectious Diseases, University of California Davis, Davis, California, USA; Department of Internal Medicine, Division of Infectious Diseases, University of California Davis, Davis, California, USA

**Keywords:** wildfire particulate matter, COVID-19, spatial autoregressive models, spatial autocorrelation

## Abstract

**Background:**

Climate change is anticipated to have profound effects on human health, including in infectious diseases. Wildfires have been increasing in frequency and intensity due to climate change and have been linked to worsening respiratory disease outcomes. We aimed to demonstrate whether there was an association between wildfire smoke and coronavirus disease 2019 (COVID-19) in California during 2020.

**Methods:**

We used an ecologic cohort study with a spatial autoregressive model to test for associations between wildfire smoke, measured as particulate matter <2.5 µg/m^3^ and COVID-19 cases and deaths at the county level in California in 2020. All data was downloaded from open sources that were freely available to the public. In our spatial autoregressive model, we adjusted for demographic, environmental factors and spatial autocorrelation that could be associated with the exposure and outcome.

**Results:**

In an adjusted analysis, we found a 1-month lag increase of 203 COVID-19 cases per 10 000 persons per 10 µg/m^3^ of smoke exposure (*P* < .001) at the county level. There was a 1-month lag increase of 2.75 COVID-19 deaths per 10 000 persons per 10 µg/m^3^ of smoke exposure (*P* < .001) at the county level. These findings were attenuated in the second month after smoke exposure, with a 2-month lag increase of 80.6 COVID-19 cases per 10 000 persons per 10 µg/m^3^ of smoke exposure (*P* = .002) and no 2-month lag association with COVID-19 deaths.

**Conclusions:**

The year 2020 was particularly strong for wildfires in California and a unique year for infectious diseases with the COVID-19 pandemic. Our findings demonstrate that wildfire smoke exposure likely increased the spread of COVID-19 and worsened the mortality rate.

## Graphical Abstract

**Graphical Abstract:**
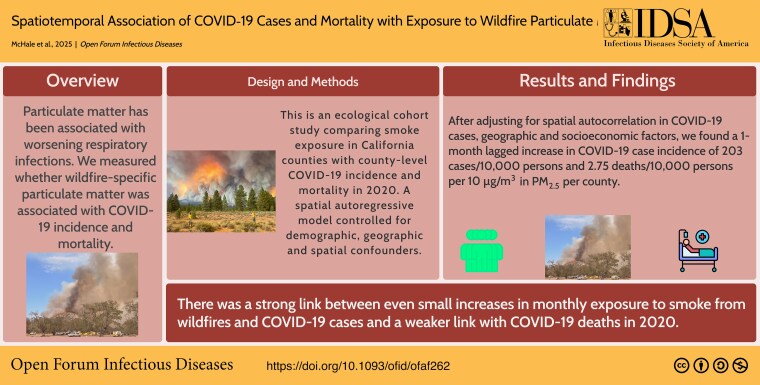
This graphical abstract is also available at Tidbit: https://tidbitapp.io/tidbits/spatiotemporal-association-of-covid-19-cases-and-mortality-with-exposure-to-wildfire-particulate-matter-in-2020-b3009a9e-97dd-427f-ae67-8a15c042dd84?utm_campaign=tidbitlinkshare&utm_source=IO

## Teaser

In 2020 wildfires in California were particularly severe as the COVID-19 pandemic emerged. Wildfire particulate matter was spatially and temporally associated with an increase in COVID-19 incidence and mortality after adjusting for environmental and demographic factors.

As the global climate continues to change, the epidemiology of infectious diseases is expected to evolve simultaneously [1, 2]. In 2023, 8 cases of autochthonously transmitted malaria were diagnosed in Florida and Texas, heralding changing ecosystems of vectors and exposures to infectious diseases [3]. Increases in wildfire frequency and smoke production—a potential infectious disease vector [4]—is expected to have long-term and severe impacts on human health [5–7]. The area burned by wildfire has increased significantly across much of the western United States since the 1980s [8], and climate change has been identified as the main driver of this trend [9, 10]. For this reason, the warming climate is likely to be associated with more wildfire acres burned and continuing significant impacts on human health from smoke [11]. Estimates in recent years indicate that the amount of fine-particle air pollution—particulate matter <2.5 µg/m^3^ (PM_2.5_) in aerodynamic diameter—accounted for by wildfire smoke has risen from 20% to 50% in the western United States, with a continuing positive trend [11].

Air pollution, in particular PM_2.5_ was shown in prior studies to be associated with increases in cardiovascular and respiratory disease related mortality and hospitalizations, and PM_2.5_ can routinely reach distal alveoli within the lungs and subsequently enter the bloodstream [12, 13]. Study findings suggest that PM_2.5_ emitted in wildfire smoke is more toxic than other atmospheric sources [14–16], and the presence of PM_2.5_ can reduce the diffusion coefficient of viruses, fungi, or other pathogens [17, 18] or serve as a “carrier” [19]. In addition, wildfire smoke exposure has been linked to various human diseases, including exacerbation of cardiovascular and respiratory disease [20–24] and in some cases, coccidioidomycosis (ie, Valley fever) [18, 25].

Severe acute respiratory syndrome coronavirus 2 (SARS-CoV-2), the causative agent for coronavirus disease 2019 (COVID-19) has also been linked to short- and long-term PM_2.5_ levels [19, 26–29] and wildfire smoke [29]. In 2020, California recorded its largest wildfire year in history with 6 “exceptionally large” wildfires contributing to >2 million hectares burned [30], coinciding with the spread of COVID-19 in a naive population. Zhou et al [29] examined correlation between 2020 daily levels of PM_2.5_ associated with remotely sensed wildfire smoke plume presence in California, Washington, and Oregon and found an excess of COVID-19 cases and deaths after exposure to wildfires, using a bayesian hierarchical model that did not account for spatial associations. This raises questions about the potential influence of spatial patterns on the resulting analysis of the interaction of wildfire-originating PM_2.5_ and patterns of COVID-19. In addition, Meo et al [28] found that there was a correlation between elevated PM_2.5_ levels and increases in COVID-19 cases and deaths in San Francisco. Wu et al [27] used an ecological analysis of overall PM_2.5_ to show an association with fine-particulate matter and COVID-19 across the United States in 2020. Ademu et al evaluated the effect of air pollution, specifically carbon monoxide and nitrogen dioxide, and also found a positive association with COVID-19 [31].

We present a new spatial autoregressive (SAR) model to account for spatial association, a key variable that affects pathogens transmitted person to person through aerosols and respiratory droplets. The previously described studies do not account for the spatial effects inherent in the person-to-person transmission of a respiratory disease. Our smoke exposure variable was developed by Childs et al [32] to specifically extract the smoke-related PM_2.5_ using a complex model described below, allowing us to isolate the association between wildfire smoke and COVID-19. Our study reveals how the wildfire smoke exposure in California during 2020 may have fanned the flame of the spread of SARS CoV-2 and affected COVID-19 outcomes.

## METHODS

### Study Design

This ecologic study uses the counties of California as the units of analysis. We determined whether there was an association between exposure to wildfire smoke PM_2.5_ and COVID-19 cases and deaths 1 and 2 months later.

### Smoke Exposure Data

Childs et al [32] recently developed a model to determine the level of PM_2.5_ attributable directly to wildfire smoke in the United States and published the data for public use. Using these data, we determined the county level PM_2.5_ smoke exposure in California. A detailed explanation of the smoke PM_2.5_ dataset is available from Childs et al [32]. In brief, the dataset was produced by first identifying smoke days based on overhead satellite imagery to identify smoke plumes. Ground-based Environmental Protection Agency monitoring stations measured deviations from location- and month-specific PM_2.5_ and on nonsmoke days to identify anomalies that could be specifically attributed to wildfire smoke [32]. Finally, they created a model that would estimate the smoke PM_2.5_ across the United States [33] and produced a county-level grid of the contiguous United States with daily PM_2.5_ attributable to smoke from 2006 to 2020 [32]. We determined the average monthly smoke PM_2.5_ calculated from the daily smoke PM_2.5_ for each county of California.

### Covariates

Temperature and precipitation data for California counties were downloaded from the National Oceanic and Atmospheric Administration (NOAA) website [34]. Administrative barriers and elevation data were downloaded from the Database of Global Administrative Areas [35].

Demographic data—including population, median income, median age, race, and occupation—were downloaded from the American Census Survey, based on the 2020 US Census [36]. Since outdoor occupations are more likely to increase an individual's exposure to smoke inhalation, we used the occupation data to determine the percentage of workers in each county whose occupation is predominantly outdoors. Occupations that were considered “outdoor” included workers aged ≥16 years who worked in farming, fishing, and forestry occupations, specifically those who worked on a farm or ranch or in an orchard or other farming, fishing, and forestry occupations [36]. In addition, the *New York Times* (*NYT*) published a national survey of mask use between 2 and 14 July 2020 that was conducted by the survey firm Dynata, with >250 000 responses nationwide [37]. This survey asked the following question: “How often do you wear a mask in public when you expect to be within six feet of another person?” [38]. In 2020–2022, Google released mobility reports that described monthly changes in mobility based on usage of certain Google products, such as Google Maps [39]. These were published as percentage changes in visits to grocery stores, pharmacies, parks, recreational areas, workplaces, and residences [39]. We averaged the percentage change in mobility for each month across these locations.

### Outcomes

We used COVID-19 data, which was aggregated by the *NYT* from state and local health departments [40], to perform an analysis of whether there was a spatiotemporal association between wildfire smoke exposure and the COVID-19 incidence and mortality rates in California in 2020. The COVID-19 data were downloaded as cumulative case and death incidence per month per county in California from the *NYT* Github [38]. We subtracted the cumulative cases and deaths for each month from each county to determine the monthly case and death incidence. We also developed an interactive Shiny app that allows the user to scroll through case and death incidence by county in California in 2020 (https://mchal053.shinyapps.io/smoke-COVID/) and an animated figure that automatically scrolls through the month of the year for our key outcomes (https://rpubs.com/tmchale/1140399).

### Statistical Analysis

We produced an unadjusted and adjusted SAR model to assess the association of wildfire smoke PM_2.5_ and COVID-19 case and death incidence in 2020 in California [41]. *Spatial autocorrelation* is the term used to describe the phenomenon by which the value of a variable in a particular location is likely to be associated with the value of the same variable in neighboring locations. Respiratory pathogens like SARS CoV-2, and environmental factors like wildfire smoke have been shown to exhibit patterns of spatial autocorrelation [42, 43].

We used a lagged case and death incidence of 1- and 2-month intervals from the month of smoke PM_2.5_ exposure. The adjusted SAR model included month of the year, average annual temperature, monthly precipitation, mean elevation in each county, demographic data (including median income, percentage of outdoor occupations, median age, and percentage of population who are white), percentage change in mobility, and reported mask use in July 2020. The SAR model is defined as *Y* = ρ*WY* + *X*β + ∈, where *Y* is the dependent variable, ρ the spatial autoregressive parameter that quantifies the strength of spatial dependency, *W* the spatial weights matrix based on inverse distance weighting of the mean distance between major cities in California, *X* a matrix of independent variables, β their coefficients, and ∈ the error term [41]. The model is determined by maximum likelihood estimation of the spatial autoregressive parameter first (ρ), and subsequent β coefficients are then determined by generalized least squares.

We calculated a log likelihood for the SAR models to estimate *P* values for overall model fit. For the SAR model, ρ was a measure of spatial autocorrelation in the model after adjusting for confounders. A large ρ indicates that the confounders do not account for all spatial autocorrelation in the dependent variable. Since the ρ statistic remained large after adjustment for confounders, we suspected that there may be unaccounted autocorrelation among the residuals. Thus, we included a spatial errors model in the Supplementary Materials, which accounts for autocorrelation among the residuals (Supplementary Tables 1 and 2).

In a sensitivity analysis, we also conducted a linear mixed effects regression model (Supplementary Tables 3 and 4). The model included smoke PM_2.5_ as a fixed effect and the random intercept for each county name to account for nonindependence of observations within the same geographic unit. For the linear mixed regression models, we used a *t* distribution to estimate *P* values and Satterthwaite's method to estimate the degrees of freedom. All statistical analyses and geographic information system maps were performed and produced using R Studio or QGIS software [44, 45].

## RESULTS

**Figure 1.**
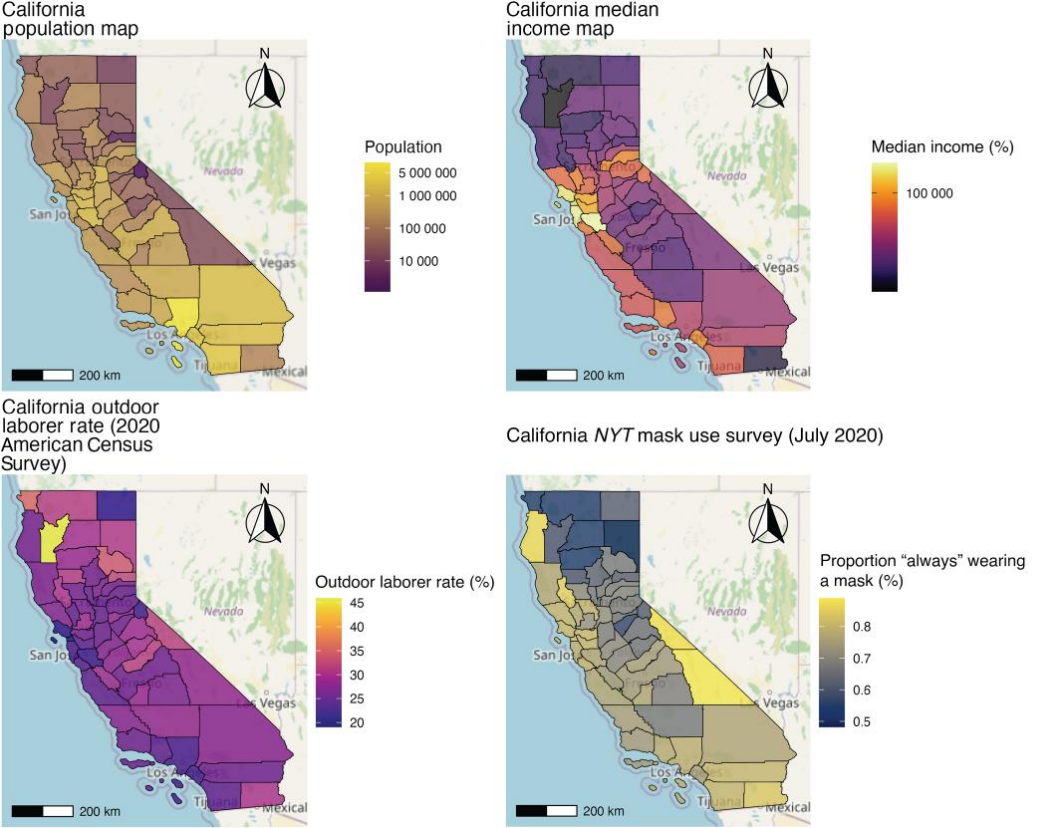
Demographic data by county in California based on the 2020 American Census Survey [36] and *New York Times* (*NYT*) mask use survey [37] conducted in July 2020. The population of California is generally larger in the southern region, especially Los Angeles County. The median income is highest in the San Francisco Bay area. The outdoor laborer rate is generally higher in Northern California, especially the rural county of Trinity. Self-reported mask use in July 2020 was high throughout the state but lowest in the northern region.

**Figure 2.**
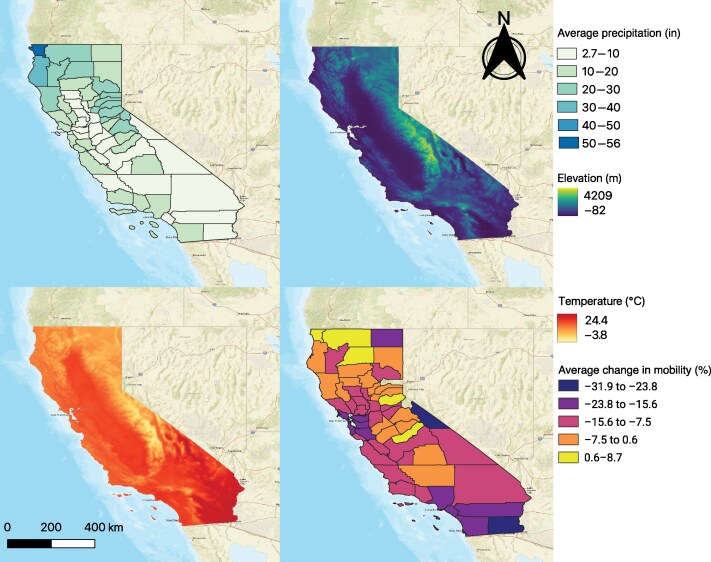
Environmental risk factors in California in 2020 based on data from the National Oceanic and Atmospheric Administration [34]. The average monthly precipitation was highest in Northern California, especially Del Norte County. The average daily temperature was highest in Southern California and lowest in the mountainous eastern region of California. Monthly mobility changed the most in Southern California counties and the San Francisco Bay area.

**Figure 3.**
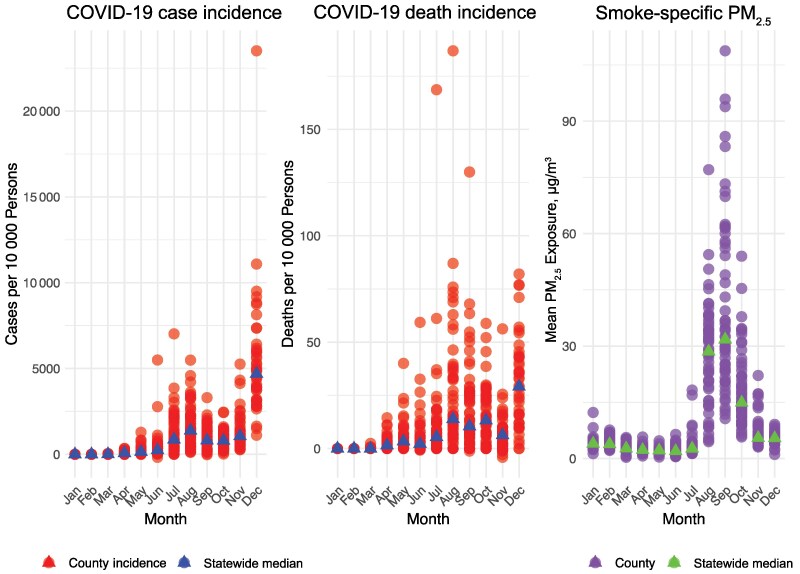
Median coronavirus disease 2019 (COVID-19) case and death incidence for each county in California and overall case and death incidence in each county and statewide county median case and death incidence are shown in the first 2 plots. Mean wildfire smoke particulate matter <2.5 µg/m^3^ (PM_2.5_) exposure (in micrograms per cubic meter) in each California county and median smoke exposure are displayed in the third plot. There was a surge in COVID-19 cases and deaths in California in the summer and winter of 2020 but a large degree of variation across counties. There was a surge in smoke exposure in the fall of 2020 and a large degree of variation across counties. See our Shiny app for interactive maps displaying these data (https://mchal053.shinyapps.io/smoke-COVID/).

California's diverse geography and demographics played a role in its complex relationship with the COVID-19 pandemic. The population of California is predominantly centered within Los Angeles County, the Southern California region, and to a smaller degree in the Northern California region near San Francisco–Oakland–San Jose (Figure 1). The highest median income is in the San Francisco Bay area, while the highest rates of outdoor laborers occurred in rural Northern California counties. The *NYT* mask use survey [37] in July 2020 reported a high degree of mask use, with a vast majority of counties reporting >50% of respondents stating they always wear a mask when in public and expecting to be within 6 feet of another person. Monthly precipitation in 2020 was highest in Northern California while average daily temperatures were highest in Southern California (Figure 2). There was a surge in cases and deaths due to COVID-19 in California in the summer months of July and August, followed by a larger surge in November and December (Figure 3). There was also a distinct increase in smoke exposure in August, September, and October. There was a large degree of variability across counties in California (Figure 3).

In an unadjusted SAR model of the confounders (Supplementary Table 5), we found that California counties had an increase in COVID-19 cases of 177/10 000 persons/1°C increase in average temperature (95% confidence interval [CI], 96.1–258). California counties had a decrease in cases of 202/10 000 persons per inch of monthly precipitation (95% CI, −289 to 117) and a decrease of 101/10 000 persons per 1-m increase in average elevation (−179 to −23.8). We found an increase in COVID-19 deaths of 4.62/10 000 persons per 1°C increase in average temperature (95% CI, 2.98–6.26), a decrease in deaths of 3.98/10 000 persons per inch of monthly precipitation (−5.62 to −2.32), and a decrease in deaths of 1.19/10 000 persons per 1-m increase in average elevation (−3.48 to −.50). We also found an increase of 2.25 COVID-19 deaths per 1% increase in county respondents reporting wearing a mask in public within 6 feet of other people (95% CI, .69–3.82).

We assessed incidence of COVID-19 by county based on association with particulate smoke exposure from wildland fires. These associations can be explored interactively in Supplementary Figures 1 and 2. In an unadjusted SAR model, we found that California counties had 1-month and 2-month lag increases in COVID-19 case incidence of 253/10 000 persons and 144 cases/10 000 persons per 10-µg/m^3^ increase in monthly average PM_2.5_ smoke exposure, respectively (both *P* < .001) (Table 1). In the same model, we found that there was a 1-month lag increase in COVID-19 deaths of 3.53/10 000 persons (*P* < .001) per 10 µg/m^3^ increase in PM_2.5_ smoke exposure, but there was no 2-month lag increase in COVID-19 deaths. A large *ρ* estimate with *P* < .001 for all models indicates that there was a strong degree of spatial autocorrelation for COVID-19 case incidence and death among California counties in 2020.

**Table 1.** Unadjusted Spatial Lag Model for Coronavirus Disease 2019 Case and Death Incidence in California Counties in 2020

Duration of Lag	Incident Cases/10 000 Persons^a^ (95% CI)	*P* Value	ρ Estimate (95% CI)	*P* Value	Incident Deaths/10 000 Persons^a^ (95% CI)	*P* Value	ρ Estimate (95% CI)	*P* Value
1 mo	253 (202–303)	<.001	0.368 (.259–.477)	<.001	3.53 (2.52–4.53)	<.001	0.430 (.329–.531)	<.001
2 mo	144 (90.2–197)	<.001	0.322 (.203–.441)	<.001	0.503 (−.60 to 1.61)	.37	0.379 (.268–.490)	<.001

Abbreviation: CI, confidence interval.

^a^Per 10-µg/m^3^ increase in smoke exposure to particulate matter <2.5 µg/m^3^. We found very strong evidence for a 1-month lag increase in coronavirus disease 2019 (COVID-19) cases and deaths and a 2-month lag increase in cases; there was no 2-month lag increase in COVID-19 deaths.

We assessed whether the risk of smoke exposure for COVID-19 was consistent when adjusting for other factors potentially associated with COVID-19. In an adjusted SAR model, we controlled for median income, outdoor laborer rate, median age, race, percentage of monthly change in mobility, average 2020 temperature per county, monthly 2020 precipitation per county, average elevation, month of the year, and percentage of respondents reporting “always” wearing a mask when in public within 6 feet of other another person (Table 2). We found that California counties still had 1- and 2-month lag increases in COVID-19 cases of 203/10 000 persons and 80.6/10 000 persons per 10 µg/m^3^ increase in PM_2.5_ smoke exposure, respectively (both *P* < .001) (Table 2).

**Table 2.** Adjusted Spatial Lag Model for Coronavirus Disease 2019 Case and Death Incidence in California Counties in 2020^a^

Duration of Lag	Incident Cases/10 000 Persons^b^ (95% CI)	*P* Value	ρ Estimate (95% CI)	*P* Value	Incident Deaths/10 000 Persons^b^ (95% CI)	*P* Value	ρ Estimate (95% CI)	*P* Value
1 mo	203 (154–251)	<.001	0.238 (.109–.367)	<.001	2.75 (1.65–3.85)	<.001	0.202 (.06–.341)	.005
2 mo	80.6 (30.0–131)	.002	0.095 (−.056 to .244)	.22	−0.635(−1.75 to .48)	.26	0.165 (.022–.309)	.02

Abbreviation: CI, confidence interval.

^a^The model is adjusted for median income, outdoor laborer rate, average 2020 temperature per county, monthly 2020 precipitation per county, average elevation, month of the year, percentage of population who are white, median age, monthly change mobility change, and percentage of respondents reporting “always” wearing a mask when in public within 6 feet of other another person. We found very strong evidence for 1-month lag increases in coronavirus disease 2019 (COVID-19) cases and deaths and a 2-month lag increase of in COVID-19 cases; there was no 2-month lag increase in COVID-19 deaths.

^b^Per 10-µg/m^3^ increase in smoke exposure to particulate matter <2.5 µg/m^3^.

After controlling for possible confounders, there was 1-month lag increase in COVID-19 deaths of 2.75/10 000 persons (*P* < .001) but no 2-month lag increase in deaths. After adjustment for confounders, the *ρ* estimate was still large, at *P* < .001 for the 1-month COVID-19 case incidence, which indicates that there was spatial autocorrelation in COVID-19 case incidence not accounted for by the confounders. To fully evaluate for spatial autocorrelation, we included a spatial errors model as a sensitivity analysis (Supplementary Tables 1 and 2). That model found a similar association between smoke exposure and COVID-19 case and death incidence. After adjustment for confounders in the spatial errors model, there remained a strong degree of spatial autocorrelation in the residuals (Supplementary Table 2). These findings indicate a high degree of spatial autocorrelation for COVID-19 case and death incidence that cannot be explained by the confounding factors we adjusted for.

We also performed a sensitivity analysis using a linear mixed effects model to assess the impact of smoke exposure on 1-month and 2-month lagged COVID-19 case and death incidence. The findings from the unadjusted and adjusted linear mixed effects model were similar to those from the SAR models (Supplementary tables 3–4).

## DISCUSSION

In this ecologic cohort study using counties as the units of analysis, we found a strong association of increased COVID-19 case and death incidence in California counties in 2020 after exposure to increased levels of wildfire smoke PM_2.5_ pollution. In our unadjusted analysis there were 1-month lag increases in COVID-19 cases and deaths of 253/10 000 persons and 3.53/10 000 persons, respectively, per 10 µg/m^3^ of smoke-specific PM_2.5_. We also found strong evidence for a smaller 2-month lag increase in incidence of COVID-19 cases of 144/10 000 persons, but the mortality rate was not increased in the second month after smoke exposure. Our findings were similar after controlling for median income, average temperature, monthly precipitation, average elevation, outdoor laborer rate, month of the year, and self-reported mask usage when in public within 6 feet of another person in July 2020.

A study that used a different approach to estimate increases in wildfire smoke-specific PM_2.5_ levels found an excess of COVID-19 case and deaths in California, Oregon, and Washington in 2020 due to smoke exposure [29]. Zhou et al first defined wildfire days using the NOAA Hazard Mapping System satellite imagery-based delineation of smoke polygons and, to be conservative, selected only “heavy” smoke days based on opacity as wildfire days [29]; the PM_2.5_ considered attributable to wildfires was then estimated using counterfactual approach. We improved on this approach by using the results from Childs et al [32], which included continuous PM_2.5_ values for *all* smoke days the using NOAA Hazard Mapping System, corroborated smoke by tracking smoke to the source wildfires, used air packet trajectory models to fill gaps due to cloud-imagery interference, and assessed Environmental Protection Agency pollutant monitoring stations' anomalous PM_2.5_ levels compared with 3-year median values on nonsmoke days. The wildfire smoke exposures explored here are therefore more spatially comprehensive and sensitive to any addition of PM_2.5_ from wildfires during the study period. In addition, our use of a spatial autoregressive model is a more appropriate method of analysis for the highly spatially autocorrelated factors of both smoke exposure and SARS-CoV-2 dispersal.

Our results provide strong evidence that even a small increase in wildfire smoke attributed PM_2.5_ occurring in the western United States in 2020 affected the incidence and severity of COVID-19 in California. A plausible mechanism for this association can be built from prior air pollution studies on human health. Numerous studies have shown that exposure to pollutants in the air is associated with negative health outcomes [6, 7, 12, 13]. Furthermore, wildfire smoke has been shown to promote local oxidative stress and inflammation in the lungs [46–48]. Other studies have linked PM_2.5_ exposure specifically to lower respiratory infections, including pneumonia, bronchitis and influenza [46, 49, 50]. Inhalation of PM_2.5_ has been shown to affect the immune response by impairing mucociliary clearance, altering receptors required for viral entry, changes to antiviral interferon production, prevention of macrophage uptake and increase in viral spread through epithelial injury [51]. It is thus plausible that population-wide exposure to elevated levels of PM_2.5_ due to wildfires would decrease the infectious dose needed for SARS-CoV2 infection and predispose the lungs to further inflammatory damage by COVID-19, increasing the mortality rate.

Given the distinct association of smoke exposure with COVID-19 in a naive population, these findings provide important insights for future respiratory infections. If further studies prove this association to be causal, the increasing number of large wildfires and increasing length of the wildfire season expected due to climate change could elevate the risk and severity of respiratory infections in the population in general and increase the susceptibility to future novel respiratory infections.

While our study does control for demographic and environmental factors, we may be limited by the inability to adequately control for all confounding factors. Our univariate analysis of the confounding factors shows that there was a strong association with COVID-19 cases, deaths, or both among our chosen confounders. However, the ecologic approach and use of a single state with 58 counties limits the number of variables that we can meaningfully control for in the SAR model. We did not control for some factors known to be associated with COVID-19 case and death incidence, such as race, ethnicity, and baseline mortality rate in the county. We did include month of the year as a linear covariate in our adjusted model. Despite this, we still found a strong association of smoke PM_2.5_ and COVID-19 cases and deaths at the county level in California. This approach reduces the likelihood that the surge in COVID-19 was coincidental with the peak incidence of smoke exposure in 2020. However, to rule out this possibility, further studies using smoke-specific PM_2.5_ and COVID-19 are needed from multiple years.

The COVID-19 data relied on reporting of local health departments, which were at times overwhelmed during 2020, leading to delays in reporting. Reporting delays would lead to case numbers in a given month actually reflecting case incidence of prior months, which might affect the association with smoke inhalation. However, our use of an SAR and mixed-effects model would account for variation at the county level, minimizing the effect of large outliers that may be caused when corrections occur in a given month or county. Our use of health department data also did not include variant type, which prevents us from analyzing whether the association with smoke exposure was enhanced or diminished by changing viral dynamics. The *NYT* mask use survey, conducted early in the pandemic, was likely heavily influenced by reporter bias and social desirability bias [52]. This may have overestimated the number of people wo were actually wearing masks in public and would not reflect behavior throughout 2020. These limitations may have been reflected in our finding that higher reported mask use was associated with increased COVID-19 mortality rate, which may be a phenomenon of cities having a higher burden of COVID-19 even while having higher mask use.

Our study demonstrates that there was a strong link between even small increases in monthly exposure to smoke from wildfires and COVID-19 cases and a weaker link with COVID-19 deaths in 2020. In addition to the COVID-19 pandemic, 2020 was a particularly intense year for wildfires, especially in California. These data, in combination with previously published works demonstrating similar findings, show that the 2020 wildfires likely enhanced the spread and severity of COVID-19. Mounting data indicate that wildfire smoke is likely to continue affecting large portions of the North American population in the coming years, and this will carry a significant burden in respiratory morbidity. Further study is needed to understand the underlying cause of this association and what measures individuals can take to mitigate their risk of smoke inhalation and the consequent adverse health outcomes.

## Supplementary Material

ofaf262_Supplementary_Data

## Supplementary Data

Supplementary materials are available at *Open Forum Infectious Diseases* online. Consisting of data provided by the authors to benefit the reader, the posted materials are not copyedited and are the sole responsibility of the authors, so questions or comments should be addressed to the corresponding author.

## Notes

***Acknowledgments.*** The study was conceived by T. C. M., D. R. B., L. K., and G. R. T. All authors contributed to the design. T. C. M. performed the statistical analyses and produced the figures and interactive apps. K. S., L. K., and J. Z. M. provided insight and review of the statistical analysis. D. R. B., L. K., and G. R. T. provided supervision. The manuscript was written by T. C. M. All authors edited and revised the manuscript and agree with publication. Large language models, specifically ChatGPT version 4.0, were used to troubleshoot code for data cleaning and preparation for statistical analysis and for troubleshooting the code for the Shiny App.

***Data availability.*** All code and datasets are available online at T. C. M.'s GitHub: https://github.com/tcmchale/COVID-california.

***Financial support.*** This work was supported by the National Institute of Allergy and Infectious Diseases (grant T32AI055433).
