# Supplementary material for: Spatiotemporal Association of Coronavirus Disease 2019 Cases and Deaths With Exposure to Wildfire Particulate Matter in 2020
Source: Open Forum Infect Dis. 2025 Jun 11;12(6):ofaf262. doi: 10.1093/ofid/ofaf262 (PMC12152477; doi:10.1093/ofid/ofaf262)
Supplement: ofaf262_Supplementary_Data [file ofaf262_supplementary_data.docx]

**Supplemental Materials**

**Supplemental Text**

Supplemental Methods

The spatial errors model adjusts for spatial dependence in the error term and is defined as: $y= X\beta+ \epsilon$ where $\epsilon= \lambda W\epsilon+u$. Y is a vector of the dependent variable, X is a matrix of independence variables, $\beta$is a vector of regression coefficients, and $\epsilon$is a vector of spatially autocorrelated error terms. The $\epsilon$ equation is defined by $\lambda$, which is the spatial error parameter capturing the degree of spatial autocorrelation in the error terms, W is a spatial weights matrix based on inverse distance weights of mean distance between major cities in California and *u* is a vector of random errors.

**Supplemental Figure 1: Screenshot of the Shiny app demonstrating COVID-19 case and death incidence each month in California** on the top map and smoke exposure on the bottom map. On the left-hand side are controls to toggle between COVID-19 case and death maps for the top map and then below are selectors for selecting the month for COVID-19 case or death and then fore smoke exposure. Link: <https://mchal053.shinyapps.io/smoke-covid/>


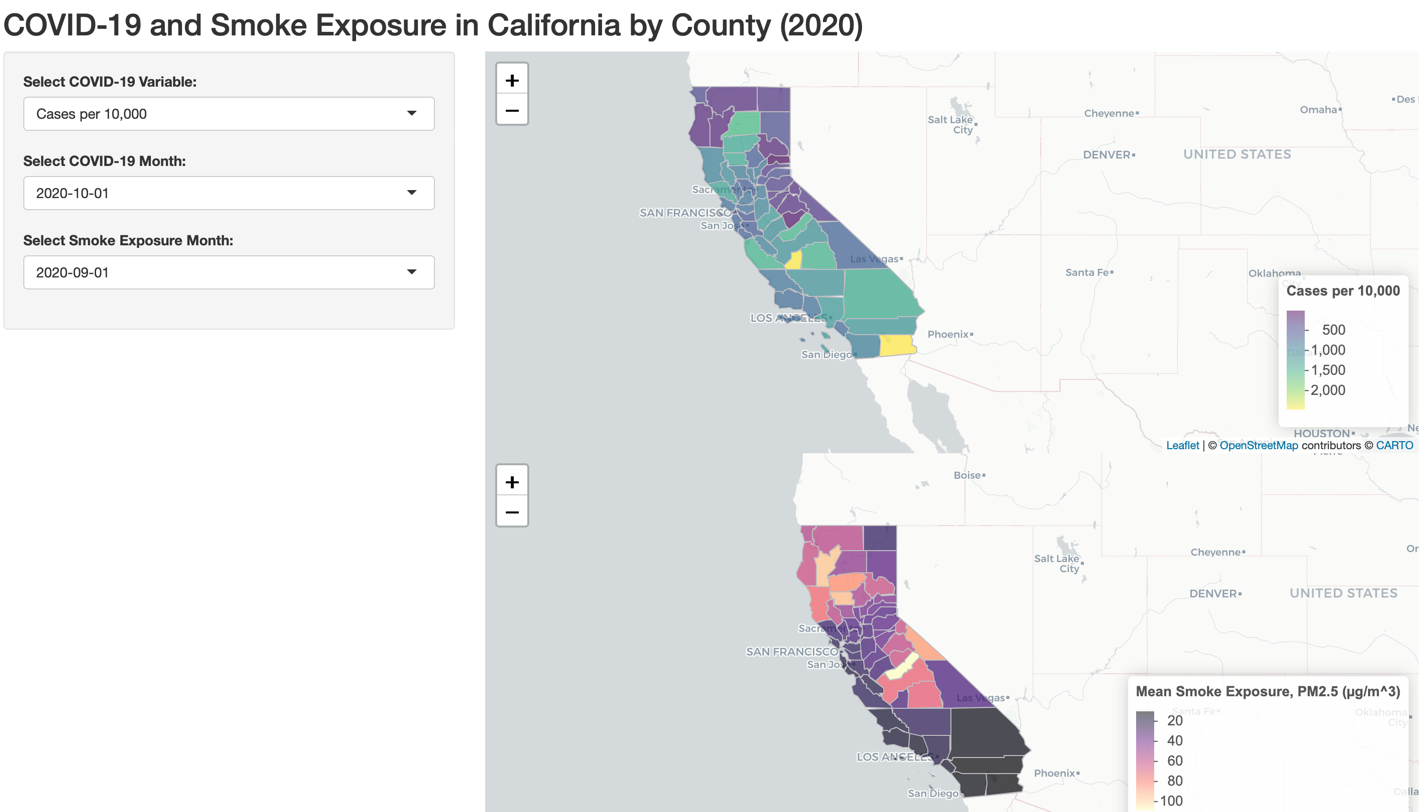


**Supplemental Figure 2: Animated GIFs of monthly COVID-19 cases, deaths and smoke PM_2.5_ exposure by county.** Link: <https://rpubs.com/tmchale/1140399>


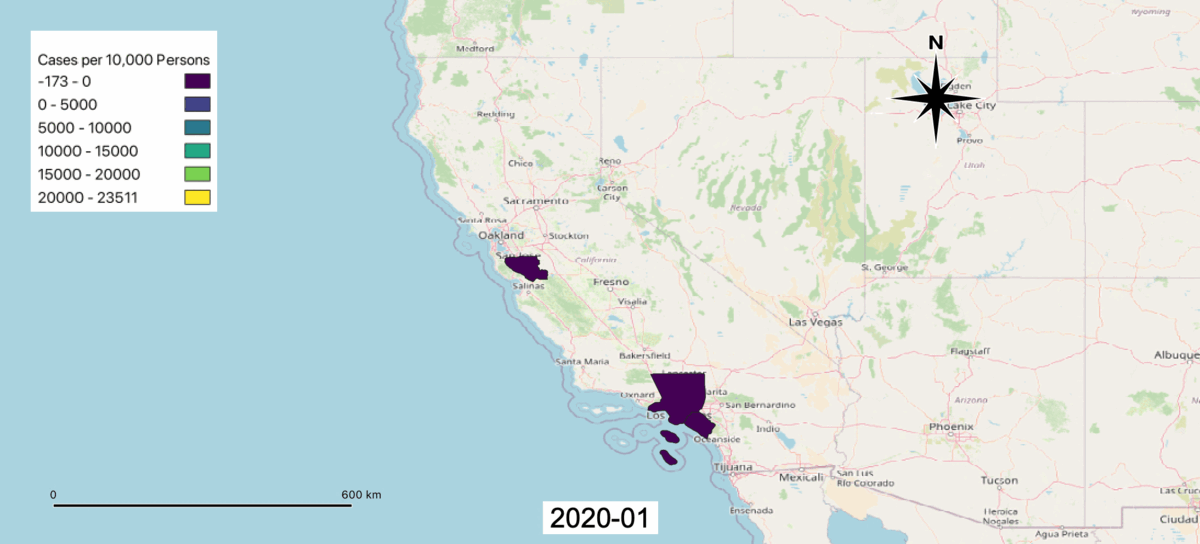


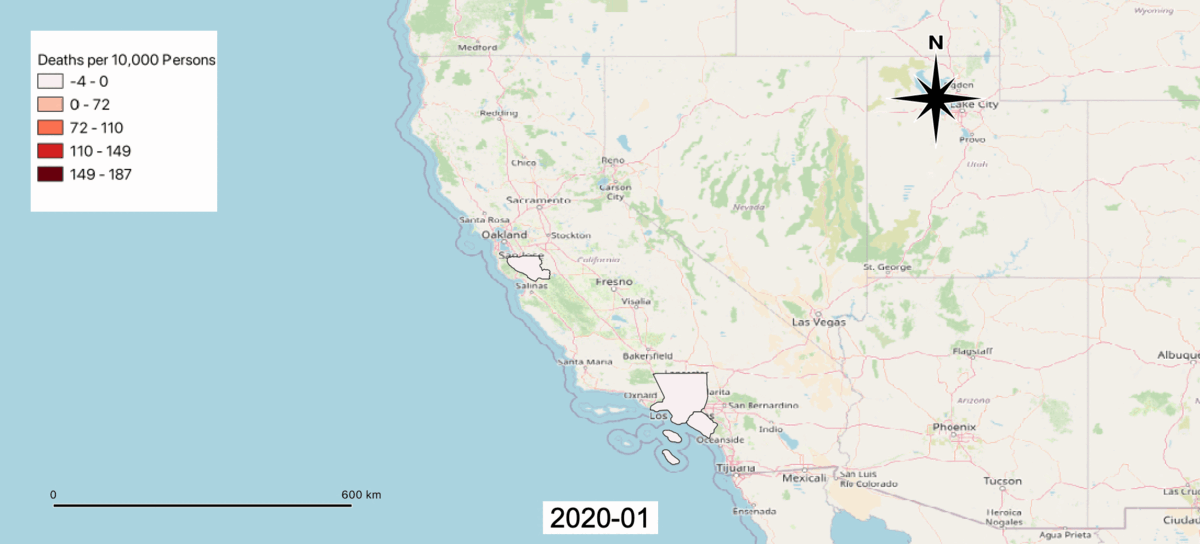


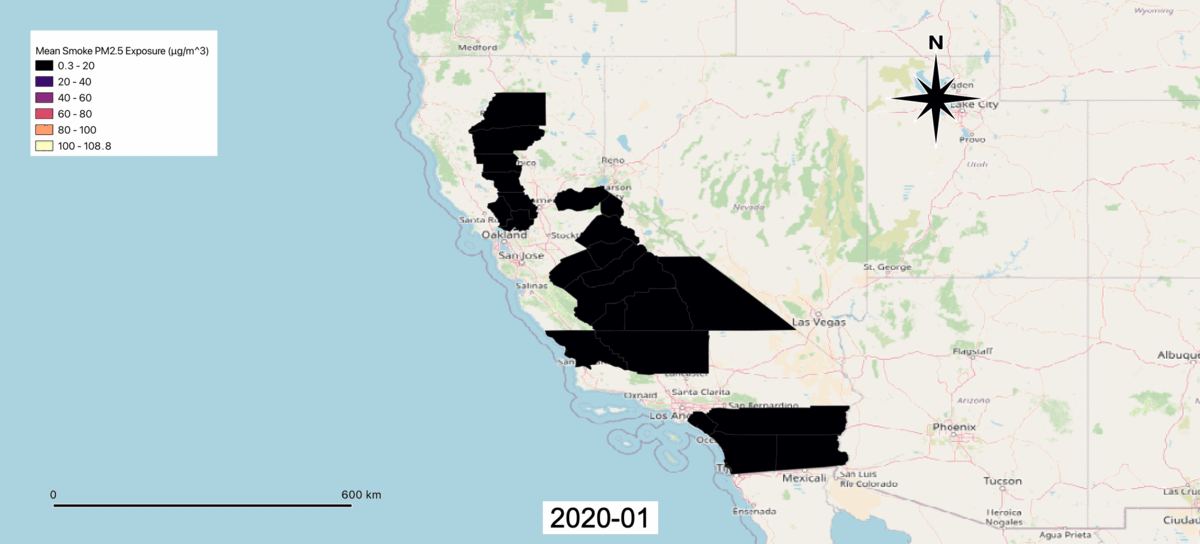


**Supplemental Table 1: Unadjusted spatial errors model for COVID-19 case and death incidence** per 10,000 persons in California counties per 10µg/m^3^ increase in PM_2.5_ smoke exposure.

|  | **One-Month Lag** | | | |
| --- | --- | --- | --- | --- |
|  | **Number of Incident Cases (95% CI)** | ***P*** | **Number of Incident Deaths (95% CI)** | ***P*** |
| **Smoke Exposure** | 264 (215, 313) | < 0.001 | 3.74 (2.76, 4.71) | < 0.001 |
|  | $\lambda$ estimate = 0.40  95% CI = (0.29, 0.51)  p-value < 0.001 | | $\lambda$ estimate = 0.45  95% CI = (0.35, 0.55)  P < 0.001 | |
|  | **Two-Month Lag** | | | |
|  | **Number of Incident Cases (95% CI)** | ***P*** | **Number of Incident Deaths (95% CI)** | ***P*** |
| **Smoke Exposure** | 154 (101, 206) | < 0.001 | 0.66 (-0.43, 1.75) | 0.236 |
|  | $\lambda$ estimate = 0.342  95% CI = (0.22, 0.46)  p-value < 0.001 | | $\lambda$estimate = 0.38  95% CI = (0.27, 0.49)  p-value < 0.001 | |

**Supplemental Table 2: Adjusted spatial errors model for COVID-19 case and death incidence per 10,000 persons per 10 µg/m^3^ increase in PM_2.5_ smoke exposure.** Controlled for median income, outdoor laborer rate, average 2020 temperature, cumulative 2020 precipitation, month of the year, percent of population who is white, median age, percent change in mobility, and percent of survey respondents who “Always” wore a mask when in public contact with people within 6 feet in July, 2020.

|  | **One-Month Lag** | | | |
| --- | --- | --- | --- | --- |
|  | **Number of Incident Cases (95% CI)** | ***P*** | **Number of Incident Deaths (95% CI)** | ***P*** |
| **Smoke Exposure** | 204 (156, 251) | < 0.001 | 2.72 (1.64, 3.80) | < 0.002 |
|  | $\lambda$ estimate = 0.283  95% CI = (0.15, 0.41)  P < 0.001 | | $\lambda$ estimate = 0.22  95% CI = (0.08, 0.36)  P < 0.001 | |
|  | **Two-Month Lag** | | | |
|  | **Number of Incident Cases (95% CI)** | ***P*** | **Number of Incident Deaths (95% CI)** | ***P*** |
| **Smoke Exposure** | 82.3 (32.6, 132) | 0.002 | -0.67 (-1.39, 0.55) | 0.230 |
|  | $\lambda$ estimate = 0.18  95% CI = (0.038, 0.32)  P = 0.016 | | $\lambda$ estimate = 0.20  95% CI = (0.06, 0.35)  P = 0.005 | |

**Supplemental Table 3**: **Unadjusted linear mixed regression model for COVID-19 case and death incidence per 10 µg/m^3^ increase in PM_2.5_ smoke exposure.**

|  | **Case Incidence per 10,000 Persons (95% CI)** | **P** | **Death Incidence per 10,000 Persons (95% CI)** | **P** |
| --- | --- | --- | --- | --- |
| **Smoke Exposure, One-month lag** | 265 (215, 313) | < 0.001 | 3.75 (2.76, 4.74) | < 0.001 |
| **Smoke Exposure, Two-month lag** | 153 (101, 206) | < 0.001 | 0.69 (-0.41, 1.79) | 0.218 |

**Supplemental Table 4: Adjusted linear mixed regression model for COVID-19 case and death incidence per 10 µg/m^3^ increase in PM_2.5_ smoke exposure.** Controlled for median income, outdoor laborer rate, average 2020 temperature, cumulative 2020 precipitation, month of the year, percent of population who is white, median age, percent change in mobility, and percent of survey respondents who “Always” wore a mask when in public contact with people within 6 feet in July, 2020.

|  | **Case Incidence per 10,000 Persons**  **(95% CI)** | **P** | **Death Incidence per 10,000 Persons**  **(95% CI)** | **P** |
| --- | --- | --- | --- | --- |
| **Smoke Exposure, One-month lag** | 207 (157, 252) | < 0.001 | 2.75 (1.66, 3.83) | <0.001 |
| **Smoke Exposure, Two-month lag** | 83.6 (32.1, 132) | 0.0021 | -0.65 (-1.75, 0.45) | 0.250 |

**Supplemental Table 5: Unadjusted spatial autore gressive model for confounders for one-month and two-month lag COVID-19 case and death incidence** per unit increase of each variable. We found strong evidence that average temperature, monthly precipitation, and mean elevation were associated with COVID-19 case incidence; while median income, average temperature, monthly precipitation, mean elevation, and reported mask use were associated with COVID-19 death incidence. There was a strong positive linear association for COVID-19 case and death incidence for each successive month 2020.

|  | **One-Month Lag** | | | |
| --- | --- | --- | --- | --- |
| **Variable** | **Case Incidence per 10,000 Persons**  **(95% CI)** | **P** | **Death Incidence per 10,000 Persons (95% CI)** | **P** |
| **Median Income, $1000** | -45,032 (-125,754; 35,688) | 0.274 | -866 (-2425, -692) | 0.276 |
| **Outdoor Laborer Rate, %** | 2.74 (-76.6, 82.0) | 0.946 | -0.203 (-1.74, 1.33) | 0.795 |
| **Average 2020 Temperature, °C** | 177 (96.1, 258) | <0.001 | 4.62 (2.98, 6.26) | <0.001 |
| **Monthly 2020 Precipitation, in** | -202 (-289, -117) | <0.001 | -3.98 (-5.62, -2.32) | <0.001 |
| **Mean Elevation, meters** | -101 (-179, -23.8) | <0.001 | -1.99 (-3.48, -0.50) | 0.009 |
| **Respondents “Always” wearing a mask, %** | 51.1 (-28.2, 130) | 0.207 | 2.25 (0.69, 3.82) | 0.005 |
| **Month of the Year** | 167 (146, 188) | <0.001 | 2.16 (1.71, 2.61) | <0.001 |
| **Percent White Alone** | -147 (-230, -63.5) | <0.001 | -2.27 (-3.85, -0.69) | 0.005 |
| **Median Age (Years)** | -267 (-355, -178) | <0.001 | -2.48 (-4.05, -0.91) | 0.002 |
| **Average Percent Change in Mobility** | -126 (-204, -48.7) | 0.001 | -2.28 (-3.80, -0.75) | 0.003 |
|  | **Two-Month Lag** | | | |
| **Median Income, $** | -22,620 (-105,923; 60,683) | 0.595 | -886 (-2564, 794) | 0.301 |
| **Outdoor Laborer Rate, %** | -15.0 (-95.1, 65.1) | 0.714 | -0.27 (-1.88, 1.34) | 0.739 |
| **Average 2020 Temperature, °C** | 220 (134, 307) | <0.001 | 4.97 (3.19, 6.75) | <0.001 |
| **Monthly Precipitation, in** | -213 (-308, -118) | <0.001 | -2.52 (-4.45, -0.60) | 0.010 |
| **Mean Elevation, meters** | -138 (-221, -55.8) | 0.001 | -2.06 (-3.70, -0.43) | 0.013 |
| **Respondents “Always” wearing a mask, %** | 98.3 (17.1, 179) | 0.018 | 2.68 (1.01, 4.34) | 0.002 |
| **Month of the Year** | 182 (158, 207) | <0.001 | 3.01 (2.48, 3.54) | <0.001 |
| **Percent White Alone** | -175 (-261, -89.1) | <0.001 | -2.66 (-4.35, -0.96) | 0.002 |
| **Median Age (Years)** | -300 (-391, -208) | <0.001 | -2.62 (-4.26, -0.97) | 0.002 |
| **Average Percent Change in Mobility** | -88.3 (-173, -3.68) | 0.041 | -1.76 (-3.47, -0.06) | 0.042 |
